# Optimized cardiac CEST MRI for assessment of metabolic activity in the heart

**DOI:** 10.1186/1532-429X-18-S1-O70

**Published:** 2016-01-27

**Authors:** Zhengwei Zhou, Yuhua Chen, Yibin Xie, Christopher T Nguyen, Mu Zeng, James Dawkins, Zhanming Fan, Eduardo Marbán, Debiao Li

**Affiliations:** 1 grid.50956.3f0000000121529905Biomedical Imaging Research Institute, Cedars-Sinai Medical Center, Los Angeles, CA USA; 2 grid.19006.3e0000000096326718Department of Bioengineering, University of California, Los Angeles, Los Angeles, CA USA; 3 grid.25879.310000000419368972Department of Computer and Information Science, University of Pennsylvania, Philadelphia, PA USA; 4 grid.50956.3f0000000121529905Heart Institute, Cedars-Sinai Medical Center, Los Angeles, CA USA; 5 grid.24696.3f000000040369153XDepartment of Radiology, Beijing Anzhen Hospital, Capital Medical University, Beijing, China

## Background

It has been previously shown that cardiac dysfunction is associated with myocardial ATP loss. The synthesis of myocardial ATP involves the conversion of phosphocreatine to creatine catalyzed by creatine kinase. CEST has been used to map creatine distribution in the myocardium to assess metabolic activity in animals [1]. However, the previous approach requires lengthy scan time (50 min), which needs to be reduced considerably for human application.

In this work, we developed an optimized cardiac CEST technique with dramatically shortened scan time (by 10-fold), improved motion registration and CEST signal calculation, and tested its feasibility to detect chronic myocardial infarction in porcine model and also in a patient for the first time. LGE imaging was used as reference.

## Methods

**Figure 1:**
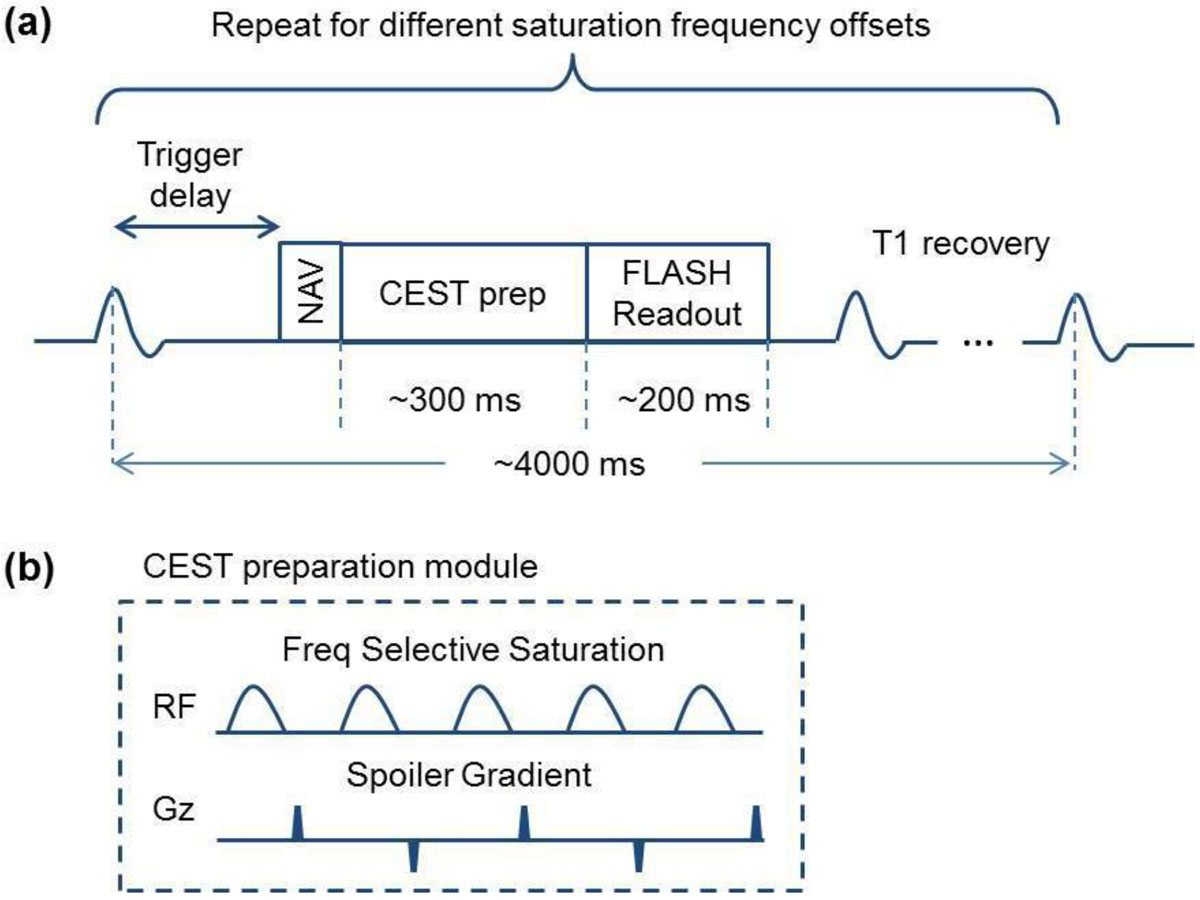
**Pulse sequence diagram of the optimized cardiac CEST imaging technique**. **(a)** ECG trigger delay is set so that the readout is in the quiescent phase of the cardiac cycle. TR is set to be 4000 ms so that another data acquisition won't start until magnetization is almost back to equilibrium. 33 images were collected at different saturation frequency offsets ranging from -4.8 ppm to 4.8 ppm with a step size of 0.3 ppm. **(b)** CEST preparation module consists of five Gaussian pulses of flip angle 2700° and duration of 30 ms at duty cycle of 50% (B1rms is 3.76uT). There is a spoiler gradient after each Gaussian pulse to crush the residual transverse magnetization.

Fig. 1 shows the pulse sequence diagram of the proposed cardiac CEST technique. ECG triggering and navigator gating were used to reduce the effects of cardiac and respiratory motion. Each image was acquired by single-shot FLASH (~200 ms readout period) with TR of 4000 ms. CEST contrast map was generated using pixel-by-pixel Z-spectrum fitting. Spatial resolution was maintained at 2.3 × 2.3 × 8.0 mm^3^. Cardiac CEST imaging technique was optimized in the following aspects: (a) Images were acquired by single-shot FLASH instead of segmented acquisition, resulting in an imaging time of 4-5 min, depending on the navigator acceptance rate. (b) All images were registered using ANTs [2] to further reduce the effect of respiratory motion. This helps make the cardiac CEST technique more robust. (c) Z-spectrum was fitted to the Lorentzian-shaped 3-pool-model to generate CEST contrast map.

Four female Yucatan porcine and one patient with chronic myocardial infarction were studied on a 3T Siemens Verio clinical scanner. LGE images were acquired as reference for myocardial infarction.

## Results

**Figure 2:**
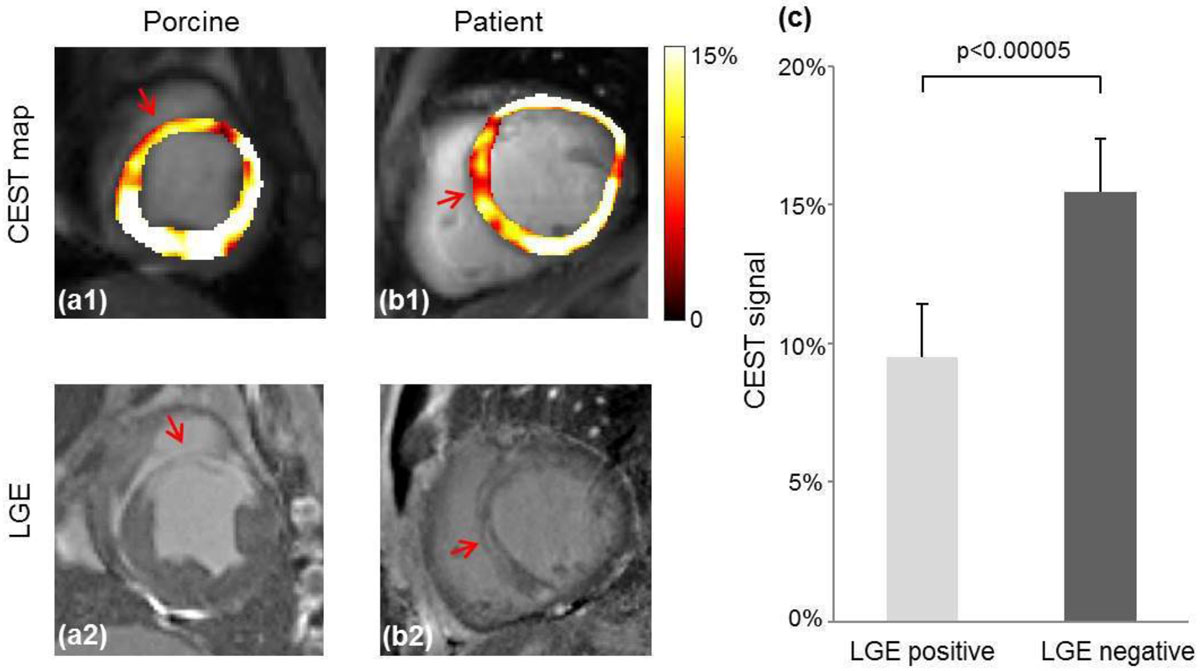
**Representative CEST contrast maps and corresponding LGE images in porcine and the patient**. (a,b) They hypointense regions (arrows) in the CEST contrast map match the LGE positive regions (arrows), suggesting scar has reduced metabolism compared to healthy myocardium tissue. **(c)** CEST signal is significantly reduced in the LGE positive region (9.54% ± 1.90%), compared to the LGE negative region (15.45% ± 2.21%), p < 0.00005.

Fig. 2(a-b) shows representative CEST contrast maps and corresponding LGE images in porcine and the patient. The hypointense region in the CEST contrast map matches the bright area in LGE image closely, suggesting that the scar region has reduced creatine distribution and lower metabolic activity compared to healthy myocardium.

Fig. 2(c) quantitatively compares the CEST signals in the LGE positive and negative regions in base, mid and apex slices in the porcine model. The CEST signal is significantly reduced in the infarct region (9.5% ± 1.9%), compared to healthy remote myocardium (15.5% ± 2.2%), p < 0.00005. In the patient, CEST signal in the infarct region is 8.4% while that in the healthy myocardium is 16.2%.

## Conclusions

We developed a clinically feasible cardiac CEST approach and performed preliminary validation studies in porcine with chronic myocardial infarction. The study also shows the feasibility of cardiac CEST imaging in a patient, for the first time. This technique has the potential to provide information on metabolic abnormalities for cardiac diseases.
